# *Trichomonas vaginalis* in Pregnancy

**DOI:** 10.1155/S1064744994000207

**Published:** 1994

**Authors:** Sebastian Faro


Infectious Diseases in Obstetrics and Gynecology 1:257–258 (1994)
© 1994 Wiley-Liss, Inc.

### *Trichomonas vaginalis* in Pregnancy

*Trichomonas vaginalis* is a common cause of vaginitis in both the pregnant and nonpregnant patient. This protozoan is usually susceptible to metronidazole, but there is reluctance to administer this antibiotic to pregnant patients. The presence of *T. vaginalis* usually indicates that the vaginal ecosystem has become unbalanced. The microflora tends to shift from a lactobacilli-dominant ecosystem to one dominated, most likely, by anaerobes.

This imbalance in the ecosystem may be the factor that is associated with preterm labor or premature rupture of the membranes. The presence of trichomonads may not be a significant factor. In addition, the abundance of bacteria provides a food source for trichomonads, which may present an opportunity of temporary treatment in the first trimester of pregnancy.

Metronidazole remains the only treatment of *T. vaginalis* vaginitis. The use of metronidazole gel is not appropriate regardless of the trimester of pregnancy. At best, the gel would provide temporary relief; however, the gel is not effective in the treatment of *T. vaginalis* vaginitis. Moreover, it does not reach significant tissue or blood levels to treat infection of Bartholin's glands, Skene's glands, or bladder involvement.

In addition to the potential for interruption of the pregnancy, the presence of *T. vaginalis* should alert the physician to the possible presence of other sexually transmitted organisms. *T. vaginalis* may cause a severe cervicitis, which may result in vaginal bleeding. Thus, a patient with an abnormal vaginal discharge and a decrease in hydrogen concentration ($pH > 5$) should be evaluated for *T. vaginalis* as well as bacterial vaginosis.

The treatment of *T. vaginalis* in pregnancy becomes a worrisome problem. Although metronidazole has proved to be relatively safe, there is great reluctance to administer this agent to pregnant women, especially in the first trimester. Antimicrobial trials are not conducted in pregnant women for obvious reasons. Perhaps the following approach can be taken in the first trimester of pregnancy. The initial treatment can be focused on reducing the number of bacteria because they will most likely consist of gram-negative facultative anaerobes and gram-negative and gram-positive obligate anaerobes. If the whiff test is positive, than an agent such as clindamycin cream can be utilized. If the whiff test is negative, amoxicillin/clavulanate (Augmentin, Beecham Laboratories, Bristol, TN), 500 mg t.i.d. for 10 days, can be utilized. If there is strong suspicion that either *Neisseria gonorrhoeae* or *Chlamydia trachomatis* or both are present, Augmentin should be effective.

Following the completion of treatment, the patient's vaginal ecosystem should be re-evaluated. The goal is not the eradication of *T. vaginalis* but a significant reduction in associated bacteria. Once the patient enters the second trimester, oral metronidazole can be administered.

Received July 7, 1994
Accepted July 7, 1994

Editorial

The obstetrician could use assurance in the treatment of *T. vaginalis* vaginitis in pregnancy. Therefore, it would be most helpful to hear from the infectious-disease specialists regarding their suggestions or recommendations in the treatment of *T. vaginalis* vaginitis in pregnancy.

*Sebastian Faro, M.D., Ph.D.*
*Editor-in-Chief*

